# Functional identification and evaluation of massively parallel neural circuits

**DOI:** 10.1186/1471-2202-14-S1-P431

**Published:** 2013-07-08

**Authors:** Aurel A Lazar, Yevgeniy B Slutskiy, Yiyin Zhou

**Affiliations:** 1 Department of Electrical Engineering, Columbia University, New York, NY, 10027, USA

## 

Parameter estimation and its evaluation are at the core of functional identification of neural circuits. How are estimates of neural circuits impacted by neuronal biophysics and particular stimuli employed in experiments [1]? What is a suitable metric to assess the goodness of the identified circuit parameters [1,2]? How can this metric be extended from a single neuron to a circuit consisting of thousands of neurons? We report new insights obtained by asking these questions in the rigorous context of channel identification/time encoding and the massively parallel models of *biophysical *circuits of the early visual system [3,4].

Our approach builds on the observation that during an experiment, an entire neural circuit is projected onto the space of input signals, with the circuit projection intuitively determined by the way the input stimuli explore the functional characteristics of that circuit. Assuming the general outline of the circuit architecture is known, we identify the projection of the encoding circuit whose receptive fields and parameters of individual neurons are unknown. We demonstrate that the projection (i.e., each identified parameter) is stimulus-dependent and derive a quantitative description of this dependence in the *parameter space*. We provide a rigorous theoretical analysis and derive conditions under which the distance between the original and identified parameters can be made arbitrarily small.

After identification, one typically evaluates the estimated parameters by how well individual model neurons predict recorded responses [1,2]. Although such evaluation in the *spike-train *domain (space) can be useful, it is often insufficient, especially in a population encoding setting. We argue that it is beneficial to evaluate the identified circuit in the *stimulus space *by looking at the amount of information that the spike train ensemble retains about the stimulus. Specifically, by employing a nonlinear decoder, we reconstruct novel stimuli *sensed *by the circuit (*i.e.*, not necessarily the original stimuli). In contrast to linear decoders applied to firing rates of a small population of neurons [5,6], our nonlinear decoder works directly with spike times produced by thousands of neurons and is capable of high-fidelity reconstruction of the sensed stimulus. Thus, evaluation of the entire identified neural circuit is reduced to intuitive comparisons in the stimulus space, thereby augmenting the usual neuron-by-neuron comparison in the spike-train space. For images and video, such an evaluation can be visually performed.

We present extensive simulation results for massively parallel neural circuits that were identified using natural video with the aid of GPU computing. First, we show that for a faithful encoding model, high quality reconstruction of visual stimuli from spike trains of biophysical neurons is possible. The quality of the decoded video improves with the length of stimuli used in identification, implying a higher accuracy of neural circuit identification. By comparing the reconstructed stimulus with the original, we then demonstrate an algorithm for determining if additional experiments need to be carried out. Finally, we explore the use of the latter algorithm in the case of unobservable/ missing circuit outputs and as a means of exploring the circuit function.

## References

[B1] Houghton C Victor JD Measuring representational distances - the spike-train metrics approach Understanding Visual Population Codes 2011 MIT Press

[B2] Wu MC-K David SV Gallant JL Complete functional characterization of sensory neurons by system identification Annu Rev of Neurosci 2006 29 477 505 16776594 10.1146/annurev.neuro.29.051605.113024

[B3] Lazar AA Slutskiy YB Channel identification machines Comput Intell Neurosci 2012 2012 209590 23227035 10.1155/2012/209590 PMC3505648

[B4] Lazar AA Pnevmatikakis EA Video Time Encoding Machines IEEE Trans Neural Netw 2011 22 461 473 21296708 10.1109/TNN.2010.2103323 PMC3758754

[B5] Stanley GB Li FF Dan Y Reconstruction of natural scenes from ensemble responses in the lateral geniculate nucleus J Neurosci 1999 19 18 8036 8042 10479703 10.1523/JNEUROSCI.19-18-08036.1999 PMC6782475

[B6] Warland DK Reinagel P Meister M Decoding visual information from a population of retinal ganglion cells J Neurophysiol 1997 78 5 2336 2350 9356386 10.1152/jn.1997.78.5.2336

